# Supplementary material for: Microbes display broad diversity in cobamide preferences
Source: mSystems. 2025 Mar 21;10(4):e01407-24. doi: 10.1128/msystems.01407-24 (PMC12013260; doi:10.1128/msystems.01407-24)
Supplement: Table S1 — EC50 values calculated from data in Fig. 1B-J. [file msystems.01407-24-s0002.docx]

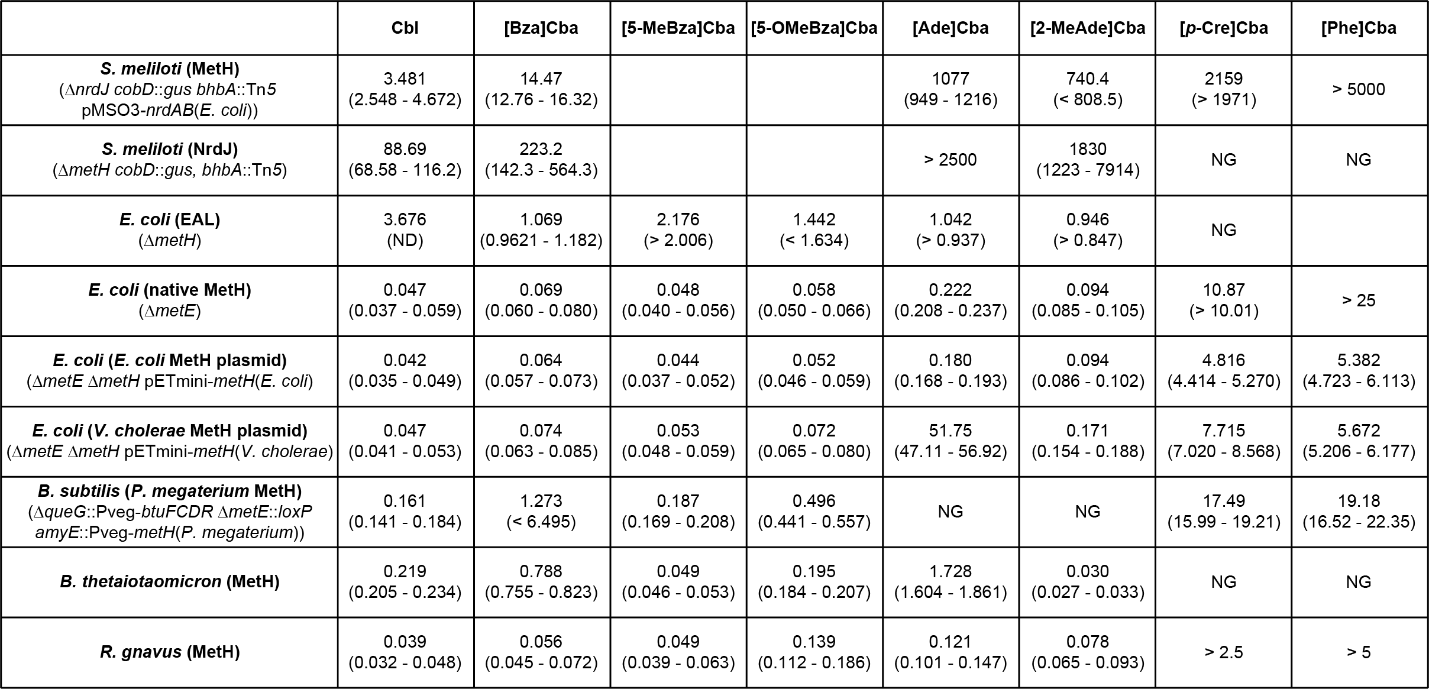


**Table S1. EC_50_ values calculated from data in Figure 1 B-J.** EC_50_ values (nM) and 95% confidence intervals (in parentheses) are given for each growth condition shown in Figure 1B-J (four-parameter non-linear fit in GraphPad Prism (v9.5.1)). Genotypes are given for engineered strains. NG, no growth; empty, not measured. A greater than (>) value for EC_50_ is an estimate for cultures that failed to reach saturation at any cobamide concentration tested. Greater than (>) and less than (<) symbols in confidence intervals were used when upper or lower bounds could not be determined, respectively; ND represents a confidence interval in which both upper and lower bounds could not be determined.
